# Which health research gets used and why? An empirical analysis of 30 cases

**DOI:** 10.1186/s12961-016-0107-2

**Published:** 2016-05-17

**Authors:** Maarten Olivier Kok, John Owusu Gyapong, Ivan Wolffers, David Ofori-Adjei, Joost Ruitenberg

**Affiliations:** Institute of Health Policy and Management, Erasmus University Rotterdam, Rotterdam, The Netherlands; VU University Amsterdam, Amsterdam, The Netherlands; School of Public Health, University of Ghana, Accra, Ghana; Department of Health Care and Culture, VU University Medical Centre Amsterdam, Amsterdam, The Netherlands; Noguchi Memorial Institute for Medical Research, University of Ghana, Accra, Ghana

**Keywords:** Research impact, Contribution mapping, Research policy, Knowledge translation, Research utilization, Priority setting

## Abstract

**Background:**

While health research is considered essential for improving health worldwide, it remains unclear how it is best organized to contribute to health. This study examined research that was part of a Ghanaian-Dutch research program that aimed to increase the likelihood that results would be used by funding research that focused on national research priorities and was led by local researchers. The aim of this study was to map the contribution of this research to action and examine which features of research and translation processes were associated with the use of the results.

**Methods:**

Using Contribution Mapping, we systematically examined how 30 studies evolved and how results were used to contribute to action. We combined interviews with 113 purposively selected key informants, document analysis and triangulation to map how research and translation processes evolved and contributions to action were realized. After each case was analysed separately, a cross-case analysis was conducted to identify patterns in the association between features of research processes and the use of research.

**Results:**

The results of 20 of the 30 studies were used to contribute to action within 12 months. The priority setting and proposal selection process led to the funding of studies which were from the outset closely aligned with health sector priorities. Research was most likely to be used when it was initiated and conducted by people who were in a position to use their results in their own work. The results of 17 out of 18 of these user-initiated studies were translated into action. Other features of research that appeared to contribute to its use were involving potential key users in formulating proposals and developing recommendations.

**Conclusions:**

Our study underlines the importance of supporting research that meets locally-expressed needs and that is led by people embedded in the contexts in which results can be used. Supporting the involvement of health sector professionals in the design, conduct and interpretation of research appears to be an especially worthwhile investment.

## Background

One of the most common laments heard in research policy circles is that the results of even the best studies are rarely translated into action [1–3]. This is especially distressing in the context of health-related research in lower-income countries, where new knowledge, well used, has the potential to save lives and improve welfare [4].

The traditional response to this apparent under-use of research is to encourage researchers to communicate their results more effectively. While it may help, better communication tends to be insufficient for improving the use of research. Communication is not ad-hoc, but requires ongoing interaction and trust, as well as relevant infrastructure [5, 6]. In addition, local capacities are required for translating generic knowledge claims to the specific local situation in which they could be useful [7–9]. An additional challenge that has long hampered research uptake in low-income countries is its limited local utility. As early as 1990, the prominent Commission on Health Research for Development reported that conventional health research contributed little to health and development in poorer countries because it was dominated by foreign scholars instead of locally embedded researchers, and met international rather than local information needs [10].

To align research more closely with national needs, local policymakers, health professionals and community representatives were encouraged to join in with NGOs, academics and others to set national health research agendas [11]. The idea was that this would lead to research driven by the demands of local stakeholders, which was more likely to be used than research driven by supply from foreign academics.

It is, however, difficult to ascertain how these various efforts influence the likelihood that research results will be used. Studies of the use of research tend to start with finalized results or evidence-based recommendations, and trace their use in action [12–16]. Most of these studies indicate that the use of research increases as potential users consider research pertinent, as research coincides with the users’ needs, as the users’ attitude is to give credibility to research and as results reach users at the right time [17–20].

In line with these observations, research funders and others have tried to foster interaction between the producers and users of research. Initially, this interaction was focused on the joint interpretation of research results and the development of recommendations. More recently, interesting methods have been designed that encourage researchers and others to think how results might be used, and engage potential users from the time research is planned and throughout research processes [21–24].

While approaches such as priority setting and involving potential users are increasingly promoted, there are few systematic studies that examine how they influence the eventual use of research [25–27]. Such studies need to examine what happens throughout research processes and relate that to the use of results [28].

Our work aimed to fill this gap, using a newly-developed method known as Contribution Mapping, to systematically assess 30 studies conducted in Ghana between 2001 and 2008 [29]. These studies were part of a program jointly developed by the governments of the Netherlands and Ghana that aimed to increase the use of research by ensuring that it was locally relevant and locally led [30]. Beginning with a national research agenda-setting process, the Ghanaian Dutch Health Research for Development Program supported research-use efforts at various points in the research process. Ghanaian professionals from three groups identified as representing the health research constituency were invited to submit research proposals that would fit the research priority agenda. These three groups comprised academia, policymakers at all levels and end-users of health research: the health workers and the communities that were to benefit from efforts to improve their health. NGOs were asked to represent the communities, and especially the more marginalized groups that were poorly reached by the regular health system. The Ghanaians leading the studies could invite Dutch researchers to collaborate with them. At the end of each study, the researchers had to submit a detailed report which contained a policy brief and specific recommendations, which were disseminated to potential key users. The research program started in 2001 and funded 79 research projects through five annual rounds of priority setting, proposal selection, funding and support.

Our study aims to map the contribution of these research projects to action and examine which features of research and translation processes were associated with the use of the results. To our knowledge, this is the first study to try to systematically analyse the relation between features of research processes and the eventual use of research across the spectrum of health research processes in a low-income country, using a substantial number of case studies.

## Methods

We used Contribution Mapping to assess how 30 research projects evolved and the results were translated into action. Contribution Mapping, which is described more fully elsewhere [29], is ground in social studies of science. Contribution Mapping recognizes that determining and attributing the ultimate ‘impact’ of research is often unrealistic and practically impossible. A true ‘impact’ perspective neglects the active role of users, who combine research outcomes with existing knowledge and use it for their own purposes in an evolving world full of ongoing processes. To take into account the active role of users and contexts, the translation of knowledge into action is better viewed as a collective process in which the agency is distributed.

A key feature of Contribution Mapping is that it contains a specific perspective on how research outcomes are integrated with existing knowledge and translated into action. This 'actor-scenario' perspective begins with the idea that those who try to translate knowledge into action put forward a more or less explicit story about a future in which they assign roles and responsibilities to a variety of ‘actors’ such as people, organizations, technologies, budgets, microbes and artefacts (e.g. these findings mean that this organization should do this, those professionals should do that, these medicine should do this, and that funder is responsible for that, etc.). Knowledge can be brought into such an ‘actor-scenario’ to confirm, support or strengthen it or introduce new elements. Knowledge can also be used to undermine the actor-scenarios of others (e.g. these findings show that they should stop funding because that policy will not work). When users bring research outcomes into such a scenario, they combine these outcomes with existing knowledge and formulate what that knowledge means for a specific aim in a specific situation. The actor-scenario perspective thus recognizes that research outcomes do not have a fixed meaning that is somehow imposed upon a passive user. While research outcomes can play a role, the perspective recognizes that such outcomes can be assigned different meanings by different actors in different situations. Regardless of the role that knowledge plays, its use can always be analyzed in terms of evolving and interacting actor-scenarios, and attempts to realize them.

Instead of trying to attribute ultimate ‘impacts’, Contribution Mapping focusses on how research and translation processes evolve and contributions to action come about, by tracing the actions of actors that are involved in, or interact with, a research project and the most likely influential users amongst them, which are referred to as potential key-users. The method follows a structured, iterative approach in which key informant interviews and document analysis are combined to develop a narrative of how processes evolved and contributions to action were realized.

### Determining whether a study was used

The outcome measure of our study was whether the results of research were used to contribute to action. A contribution to action can be described as a process in which knowledge plays a meaningful role in action for health. For the purpose of this study, we made a somewhat crude distinction between studies that were used and studies that were not. We considered a study as ‘used’ when at least one person described that produced knowledge had played a meaningful role in action for health, this was corroborated by someone else and/or documentary evidence, and the translation process seemed plausible to the external analyst. We focused on the contributions to action that could be identified between 6 and 12 months after a study was finalized. We chose this relatively fixed timeframe to allow us to compare cases.

### Case selection

For this multiple case study, we selected the first 30 research projects of the Ghanaian Dutch Health Research for Development Program that were finalized. These 30 research projects were funded between 2002 and 2004 and are described in Table 1. These research projects were all led by a Ghanaian principal investigator (PI) and included one or more co-investigators. Most research projects were completed less than 2 years after funding was provided. The research projects had budgets varying from US$ 10,000 to 20,000, excluding the salaries of the involved investigators. Until at least 6 months after a study was finalized, those involved in research and translation processes were not made aware that the use of the results would be assessed.

The research program aimed to fund research that was oriented towards the national research agenda in Ghana. The research agenda was set in four steps: (1) reviewing existing research information, (2) consulting the health sector, policymakers and NGOs about research needs, (3) interviewing community members and (4) holding a workshop to prioritize issues based upon the existence of a problem, relevance, urgency, or whether research was needed to solve the problem. The research agenda was widely disseminated and public and private research institutes, NGOs and other interested groups were invited to submit a letter of intent that fell within the research priorities. The best letters of intent were selected and research teams invited to submit a full proposal. Each proposal had to contain a section about the societal relevance/utility of the proposed research. An external, Ghanaian-Dutch scientific review committee reviewed the full proposals for scientific merit, societal relevance/utility of the research, feasibility within time, budgetary and methodological framework, and ethical considerations. Final selection of proposals was done by the Joint Program Committee based on the comments of the reviewers.

### Organization of data collection

Data collection started in March 2005 with the creation of an overview of the background and development of the research program. The assessment of each case started with reading available documentation, such as research proposals, mid-term reviews and final reports, and making a timeline-based process map. The timeline was divided into three phases: (1) formulation phase, (2) knowledge production phase and (3) the knowledge extension phase (e.g. dissemination and utilization). For each phase, the main actors, activities and interactions were mapped.

The first interview was held between 12 and 18 months after the investigators had established their results and were ready to disseminate them. The mapping process started with interviewing the principal investigators of a research project, developing a first version of the three-phase process map and identifying potential key-users and translation processes. Next, potential key-users and other informants were interviewed to trace, explore and triangulate possible contributions. In the third stage, process summaries were shared with key informants for feedback and validation. After inconsistencies were clarified, the process maps and description of contributions were finalized.

### Interviewing

One hundred and thirteen purposively selected participants were interviewed face-to-face in four rounds of data collection (2005–2008) by four different researchers; 18 participants were interviewed about several research projects and 36 participants were involved in the studies as PI or co-investigator. The others were selected as potential key-user or interviewed to further explore, triangulate or elaborate descriptions of translation processes and contributions to action. Most potential key-users had a leading role at the Ministry of Health, the Ghana Health Service or other health-related organizations.

Following the steps of Contribution Mapping, interviewees were asked to describe how the process of formulating a study proposal and conducting research had evolved, and how produced knowledge claims were disseminated and translated into action. Interviewees were encouraged to be specific about processes and interactions, to provide detailed examples and share documents that supported their claims and to provide further insight into how research and translation processes evolved. Examples of such documents are texts related to specific meetings, policy briefs, reports and presentation sheets. Emerging descriptions of translations and contributions to action were triangulated with subsequent interviewees, who could also put forward new stories of contributions and other documents that supported their claims. Interviews were audio recorded, except in five cases in which equipment failed or interviewees did not want to be recorded, and a detailed summary was made directly afterwards.

### Data management and analysis

Directly after each interview, a detailed summary was prepared. By listening to the interviews, all relevant parts were identified and transcribed verbatim. Data analysis was done in two steps: (1) a detailed qualitative within-case analysis and (2) cross-case analysis. Data analysis for each case started after the first interview and continued during the whole data collection time [28]. Interview summaries, documents and transcripts were used to iteratively develop the three-phase process maps, and the contributions to action.

To identify which features of research and translation processes were associated with the use of research, we first analyzed the individual process maps and developed a set of open codes. Examples of codes are ‘involving potential key-users in the formulation of research’ and ‘targeted dissemination of written results’. Using a constant comparative method of analysis and a manual coding system, two researchers and a research assistant then developed a more specific set of codes for those process features that seemed to matter the most [31].

We then conducted a second systematic cross-case analysis, in which, for each case, we analysed the presence and role of the selected process features and described them in a table. For each of the process features, a specific summary was developed. Our analysis was recursive, constantly moving from the specific cases, to the more general, with the aim of identifying commonalities and patterns across the variety of cases.

This study did not require ethics approval in Ghana. Under Dutch law, ethics approval in the Netherlands was also not required. Even though formal approval was not required, we followed regular ethically responsible qualitative research practice to ensure that substantive ethical issues would be dealt with in an appropriate way. Informed consent to participate in the study, record the interviews, use quotations and publish the results was obtained from all study participants. A report with the preliminary results was shared with participants in 2008. Based upon comments, two small adaptations were made in how the data was presented. The preliminary results were presented and discussed at a meeting with participants in Ghana in 2008 and at a meeting in the Netherlands in 2009. Those involved in the discussions confirmed the presented results.

## Results

We start this section with an overview of some of the studies and how they contributed to changes in health policy and practice. Next, we describe which features of research and translation processes were related to the contribution of research to action. In the last part, we further examine how translation processes evolved. In Table 1, the 30 research projects and the most prominent translation process and contributions to action are described.

**Table 1:** The 30 research projects, process summaries and identified contributions to action

Research subject and embedding	Process summary	Identified contributions to action
1. The contributing factors to high treatment defaulter rates among tuberculosis (TB) patients in Ghana Health Service (GHS) regional hospitals; data collected in hospitals with patients from villages in the region	A user-investigator played a key role in mobilizing results; one of the investigators was the head of the regional TB program and utilized the results at the regional level through this function	The results showed that financial constraint and distance were important reasons for defaulting treatment; five new treatment locations were opened and the research findings were used in a successful grant application for defaulting prevention programmes in other districts
2. The role of stigma in the spread of HIV/AIDS in a district; GHS district health administration; data collected in various communities in a rural district	The research project led to a better understanding of the role of stigma; the results were described as not directly applicable, but warranting further research	No contributions to action were identified; a new research proposal was drafted and funded
3. Resistance to anti-microbial drugs in Ghana Medical School University Dept. of Microbiology; samples collected in various hospitals in nine regions	The investigators gave technical advice about the research subject to policymakers at both the local and national level; the results were discussed with several potential key-users, which seemed to have led to their eventual use	The results showed that drug resistance was a problem in various regions; hospital laboratories received feedback about their technical performance and some initiated a training in using standardized techniques and controls for laboratory tests; the results informed discussions and decision making about essential medicines at the GHS
4. Community satisfaction, equity in coverage and implications for the sustainability of a health insurance scheme; GHS district health administration and research centre; data collected at four health centres and households in a rural district	A user-investigator played a key role in mobilizing results; one of the investigators was a regional director of the GHS, advised the committee drafting the National Health Insurance Scheme (NHIS) policy, and was involved in implementing health insurance at several levels	The study is part of a group of studies that contributed to the formulation of the NHIS policy; among others, it revealed reasons why the poorest of the poor were not enrolling; the study also provided information that was used to develop a method for identifying poor people which was used in a program that provided premium subsidies to the poorest of the poor
5. Evaluations of informal mutual health organizations in Southern Ghana; NGO involved in educational research; mutual health schemes in four districts were assessed	No potential key users were involved during the study; the finalization of the study coincided with formulation of the NHIS policy; the results and recommendations were extensively discussed with NHIS policymakers	Several recommendations were incorporated in NHIS policy; two examples are (1) providing districts with additional funding to start the NHIS in their district and (2) including the option to opt out and get involved in a personal scheme
6. What the communities think about financing health through community health insurance; GHS district health administration; study was conducted in various communities in a rural district	Based upon this research project it was recommended that health insurance should be community based; at the national level, the choice was made for a district-based health insurance scheme; no one from the national level was involved in the study	No contributions to action were identified; the community-based health insurance scheme that was set up as part of the research project is still being used 5 years after the study ended
7. The perception of and demand for mutual health insurance in a district; GHS research centre/study was conducted in various communities in a rural district	No potential key users were involved in the research processes but the research proposal and the results were discussed and interpreted together with relevant district level policymakers	The study identified existing decentralized structures that were used to improve the premium collection for health insurance; education about the insurance was intensified in areas of low enrolment that were identified
8. Communication and HIV/AIDS prevention messages through unorthodox community-based means; NGO involved in educational research; study was conducted in a rural district	The research activities involved HIV prevention, which seems to have had the desired direct impact in the local situation; no actions to use the results elsewhere were identified; the principal investigator (PI) of the study was not working in the health sector	No contributions to action were identified; there were anecdotes that board games and other educational materials were still being used at schools in the district, but his could not be confirmed
9. Assessing service delivery factors contributing to preventable maternal mortality in a region; GHS regional health administration; data were collected in 17 health facilities in a deprived region	A user-investigator played a key role in mobilizing results: one of the investigators was the regional director of the GHS, who used the results at regional level	The results were used to improve the provision and stores of consumables for obstetric care and the local drug procurement cycle; in addition, the results were used to improve the patient documentation system in a number of clinics in the region
10. Assessing the quality of immunization in a district; GHS district health administration and health research centre; data were collected in various health facilities and communities	A user-investigator played a key role in mobilizing results: one of the investigators was involved in regional and district health policy as technical advisors and regional officer for public health (including EPI) and used the results in his own work	The overall organization of the immunization in the region was improved, the communication to the communities about the immunization was improved and a policy to abolish selling food products and drugs in combination with vaccination was implemented in the region
11. Better matching the training, support and incentive systems for leaders of sub-district health teams to the requirement of the GHS at the sub-district level; GHS district administration; study in GHS metropolitan, rural and semi-urban sub-districts	A user-investigator played a key role in mobilizing results: one of the investigators was a regional director of the GHS and used the results; the results were also discussed with and sent to influential policymakers, including the deputy director general of the GHS	The research findings were used to design an initiative to strengthen sub-district-level health administrations and sub-metro-level health administrations in the Greater Accra region; discussions were initiated with the School of Public Health for a Masters of Public Health program that would be open to students without a Bachelor degree
12. Improving the quality of healthcare delivery in a district in Ghana; University Dept. of Agriculture; GHS district health administration; five health centres and communities	A user-investigator played a key role in mobilizing results: one of the investigators was a district director of the GHS and used the results; the proposal and results were discussed with the District Health Management Team and involved health institutions	As a result of the study, hospitals have established, trained and institutionalised quality assurance teams; district-wide parameters for quality assurance have been implemented; uniforms of nurses were changed to lower rivalry between regular and enrolled nurses
13. Assessing the impact of community-based health planning and services (CHPS) initiative in a district; GHS district health administration; conducted in households in a deprived rural district	A user-investigator played a key role in mobilizing results: the PI was a district director of the GHS and became the head of the nationwide CHPS program; he used the results through these functions; the proposal and results were discussed with the head of planning, monitoring and evaluation from the Ministry of Health (MOH)	The study findings were used to facilitate the nationwide implementation of the CHPS programme; the findings and experiences were used to develop a toolkit and a training programme for implementing the CHPS programme in other districts and regions
14. The contribution of public health postgraduate students’ research recommendations to districts to quality of healthcare improvement. University School of Public Health; 20 district health administrative area’s	The research revealed limited use of research from Masters of Public Health students; the project only involved university employees; no systematic changes in the organization of the research practice interface were implemented at the time	No contributions to action were identified
15. Communication channels and strategies and the potential role of community members in HIV/AIDS awareness creation and behavioural change; GHS district health administration and research centre; various communities in a rural district	The results were described as not directly applicable, but warranting further research; the project led to a proposal for an intervention study with regard to the stigmatization of HIV/AIDS patients	No contributions to action were identified; there were indications that the research project has improved the HIV education in the district but this could not be confirmed
16. Detection, assessment and prevention of adverse events following immunization with a new pentavalent vaccine; University Centre for Pharmacology; conducted in a teaching hospital, two urban polyclinics and a centre for immunization	A user-investigator played a key role in mobilizing results: the research findings were used through the roles of the investigators in policy as head of the national immunization program and as technical advisor	A multi-disciplinary advisory group to advice on AEAI was set up and continues to exist with sustainable funding from the MOH; baseline data on adverse events following immunization have been collected for the first time in Ghana to the pentavalent vaccine and will guide policymaking on immunization
17. Prevalence of HIV, hepatitis B, hepatitis C viruses, infections, TB and syphilis among prisoners in Accra; University Department of Pathology; conducted among prisoners and prison officers in three prisons in Ghana	Two investigators were influential technical advisors to health policy on various subjects (though not specifically prison health); the proposal, process and results were extensively discussed with potential key users such as the prison council, director general of the prison services, Ministry of the Interior, MOH and the parliament committee on health	Several contributions to action were identified; results played a role in the decision to include prisoners in the NHIS, provide anti-retroviral treatment and contributed to the closure of a prison that was housed in an old fortress; involved parties were advocating for more systematic medical screening of inmates and a medical facility for prisoners; these discussions were still ongoing
18. Assessment of male involvement in family planning decision-making and practice and its influence on the uptake of family planning in a district; GHS research centre and district health administration; conducted in two health centres and communities in a rural district	The research indicated that more male health workers should be trained to inform men about family planning; investigators with influence at the district and regional level were involved, but influencing the actual training of male health workers required action at national policy level, to which they had no access	No contributions to action were identified; there was little dissemination at the time of the assessment; a lack of resources for dissemination was given as the primary reason
19. An assessment of the knowledge, attitudes, beliefs and practices on HIV/AIDS as a basis for integrating prevention and care services into the CHPS in a GHS district; district health administration; conducted in 11 rural communities in isolated and deprived areas	A user-investigator played a key role in mobilizing results: one of the investigators was a district director and head of the nationwide CHPS program; he used the results in his own work through these functions	The study revealed that the perception of HIV/AIDS in the communities was little effected by CHPS; specific messages for HIV/AIDS education for within CHPS were developed and implemented in the district
20. Cost analysis and efficiency in selected hospitals in Ghana; University Department of Finance; using data from a district hospitals, mission hospitals and regional referral hospitals	The study was conducted in an academic institute; there was no substantial involvement of potential key users in the study; the results were disseminated to the administrations of three hospitals	No contributions to action were identified
21. Incidence of adverse drug reactions from anti-TB drugs among patients treated for active TB and their impact on compliance; University Department of Clinical and Social Pharmacy; 13 health facilities in two regions	Investigators gave technical advice about, amongst others, medication and TB policy; the results were presented at two academic conferences; according to the investigators, the results confirmed existing knowledge and did not imply change	No contributions to action were identified
22. Sustaining the safe motherhood clinical skills of midwives; regional health administration; health facilities and midwives spread throughout a region	A user-investigator played a key role in mobilizing results: one of the investigators was a district director and headed the safe motherhood team in the region and used the results in his own work; results were also disseminated to the health facilities and midwives involved	The research led to the identification of shortages of specific equipment and consumables necessary for safe motherhood in health facilities in the region and lapses in the safe motherhood clinical skills of midwives; the results were used to address these shortages and to provide training for midwives during supervisory visits and were discussed which the schools where midwives were trained
23. Comparative study of risk characteristics of successful and unsuccessful Mutual Health Organizations and implications for improving the success of health insurance; GHS district health administration and research centre	A user-investigator played a key role in mobilizing results: one of the investigators was involved in the development and formulation of the NHIS policy processes and stimulated the use of the findings	This research project contributed information, together with other studies, to the formulation of the district-wide-based structure of the NHIS; it also inspired the set-up of a policy advisory council in a new research project, which was to include representatives from the GHS, the National Health Insurance Council and MOH
24. Delivery of integrated pro-poor health services in the decentralized politico administrative (assembly) context; University Institute of Local Government Studies; four district assemblies	A user-investigator played a key role in mobilizing results: the study was conducted by a team from an institute that provided training, consultations, advice and support to local governments; the findings were used in the programs developed and implemented by the institute	The results were used in the design and delivery of a nationwide course for district and regional level environmental health functionaries; in addition, the results were used to shape proposals made to the Local Government Service on integrating health and local government services at the district level
25. Participation of the urban informal sector in the National Health Insurance Scheme; University Department of Medical Biochemistry; data was gathered in two urban areas known for informal trade	Involvement of potential key users, including members of the NHIS management, occurred throughout all phases of the research project; the results were discussed during a forum with the deputy director of the NHIS that was organized by the Health Research for Development Program	The results were discussed with representatives from the NHIS and were described as contributing to some reforms that were made to health insurance schemes, such an increased focus on the informal sector; though reforms were in line with the study recommendations, other factors also played a role
26. The allocative and technical efficiency of public health centres; GHS health research centre; data were gathered in multiple districts	The PI was involved in the district assembly and discussed the proposal with the district director of the GHS; there was little dissemination as the main investigator went abroad for further studies; according to the investigators, the results should be used in national level policy	No contributions to action were identified; the research project did, however, generate further interest in the area of technical efficiency such that a study on hospital efficiency was undertaken by the GHS, of which the findings were later discussed by policymakers
27. Developing unit cost data for health facilities to achieve cost standardization for an effective national health insurance scheme; GHS national headquarters	A user-investigator played a key role in mobilizing results: the PI was involved in various working groups in which national health financing policy was developed; the PI used the results to initiate and accelerate changes in financing policy; influential policymakers were engaged in interpreting and simplifying the results	The results were used in the development of the kind of flat fee system that will be used by the GHS for payments made to hospitals and health centres; the results were also used in influencing GHS hospitals funding arrangements
28. A community-based survey on the utilisation of healthcare services for gastroenteritis in children in a district; GHS health research centre; conducted in villages and health centres in a rural deprived district	The study proposal and findings were discussed with the district health administration and utilized by the investigators in the formulation and implementation of a large trial	No contributions to action were identified; the findings were used by researchers to monitor and predict the clinical attendance and behaviour of mothers seeking healthcare for their children with gastroenteritis in the district, which was a prerequisite for effective recruitment for a Rota-virus vaccination trial
29. A comparison of two approaches to increasing access and improving equity to malaria treatment among children under 5 years; GHS regional health directorate	A user-investigator played a key role in mobilizing results: one of the investigators was a regional director and advisor to the national health insurance council	The results informed the development and organization of the insurance card that was used in some districts; the results were also used to support advocacy to reduce the delay in payment to health service providers under the national health insurance scheme
30. A pragmatic randomized control trial into the compliance to artesunate-amodiaquine therapy for uncomplicated malaria in rural Ghana; GHS health research unit; data collected in a large number of communities in a rural district	A user-investigator played a key role in mobilizing results: one of the investigators gave technical advice at the district and national level related to the study subject; the study findings were discussed with the head of the national malaria control program	The study showed that patients often did not take the required dose of the therapy; education was provided to the communities through health promotion radio messages; health workers were instructed to better educate patients; a new therapy with fewer pills was under review

### The identified contributions to action

In 20 of the 30 studies, we identified a contribution to action between 6 and 12 months after the studies were finalized. We refer to these 20 studies as the ‘used’ studies.

The produced knowledge was used in many different ways. Several studies provided new knowledge about the nature and scope of health problems. This new knowledge was often used by investigators with a formal position in the health system. An example is case 1, a study into factors associated with treatment default among tuberculosis patients [32]. The PI of the study was in charge of the regional tuberculosis program and said that he initiated the study with the aim of improving tuberculosis treatment. “*For a long time I was concerned about treatment default, we talked about what to do.* […] *This study created an opportunity to do something about it, to better understand the problems and improve treatment success.*” The PI translated his results in several actions: “*This study showed that financial constraint was the main reason for patients for defaulting. Distance was one of the main issues, because they had to board vehicles to the hospital every day. When we noticed that, one of the things I have done is I have opened five new treatment locations to bring access to TB treatment. Previously there was only one treatment center in the whole district. We have also arranged for transportation money to the treatment and a daily meal during the intensive phase*.”

Another study revealed unexpected problems with the functioning and implementation of the immunization program, such as illegal charges and the sale of food supplements by health workers alongside the vaccination (case 10). Poor mothers who could not afford these extra charges and food supplements felt stigmatized and were less likely to have their children vaccinated. One of the co-investigators of the study was a district director for the Ghana Health Service, who aligned the research proposal with his concerns about the immunization program in his district and his aim to improve it. Towards the end of his study, he was promoted to the position of regional director in the Ghana Health Service. In this new function, he used the results in designing and implementing a new communication policy, a policy on abolishing illegal charges and the sale of food supplements at vaccination sites, and a new way for supervising the immunization process. “*When I started I was at district level, so I saw the need to do something to EPI* [immunization program]*. Being at regional level was a great opportunity. I met all the districts of the region and showed them the issues of immunization in Techiman, what I thought was not so different from other areas* […] *actually showing what went wrong was important for making those changes.*”

In many cases, the produced knowledge was first used in the research context and subsequently elsewhere. The co-investigator of the previous case said that he continued to use his results after he was transferred to a new region. In his new region, he informed the staff of the health districts about his study findings, encouraged them to look out for similar problems and implement the proposed policies. A district director confirmed this translation process: “*He has informed us in one of the EPI meetings* […]. *He studied the performance of the district and how to increase the performance. So he showed us the figures before the study, the difficulties they were having and after the study, the input they put in and the figures they were having. Since he came, we put everything in place.*”

In several studies, new practices, protocols and methods were developed and tested, which were first implemented locally and subsequently used elsewhere. An example is case 12, in which quality indicators were developed and teams were trained to improve the quality of care in a district. After the research project, the use of the developed indicators and quality teams was continued: “*The quality assurance has been institutionalized. Some of the district wide quality parameters that were proposed are being used already. Some others are still being reviewed for use*” (district director Ghana Health Service). A different interviewee linked to the study in case 12 described a second translation process: “*there were constraints between regular and enrolled nurses. This had been ongoing for years and came out again during the focus groups. Before the report was even finished they have changed the rule. Now they are wearing the same uniform to lower this rivalry.*” Two more examples are cases 13 and 19, in which results were used to develop a training program and support package for implementing the Community-based Health Planning and Services Initiative. The program and support package was used by different people involved in implementing this initiative throughout the country.

Interviewees also described a range of unanticipated ways in which the conduct of the research itself contributed to changes in health service practices. Case 9 provides two examples. According to one investigator: “*When we conducted the study we noticed very sharp shortages* [in consumables for preventing maternal mortality] *and linked up with the medical stores. When we found out that the stores were not there at all, we immediately reported to the regional director and made sure the situation was addressed. So indirectly that will enhance service delivery. And also the filing system: we had difficulties retrieving data. Some patients went out and with their cards. So the records were not complete. When we discovered that, we had to correct the system. So it facilitated the documentation system.*”

In several cases, results were used by different actors in different translation processes. An illustrative example is case 17, a study into the prevalence of infectious diseases among prisoners and guards in Ghana. For years, there had been anecdotes and occasional media reports about the poor health status of prisoners. After being contacted by a concerned prison officer, a university-based researcher initiated a disease surveillance study showing a significant outbreak of HIV and hepatitis C among prisoners and guards and a lot of risk behaviour among prisoners such as illegal drugs use, unprotected sex and tattooing with shared needles [33]. Counselling and treatment were provided and a peer education program was set up following the research in the prisons in which the studies were conducted. The prison service used the results to encourage the Ministry of Health to provide better health services to prisoners. Interviewees described how, after several discussions, the results played a role in the decision to include prisoners in the National Health Insurance Scheme. Other interviewees described how the results played a role in the lobby, and eventual decision to close Usher Fort prison, which was housed in an 17th century Dutch colonial fortress. Interviewees also described how the results inspired USAID to provide anti-retroviral treatment at a clinic next to Nsawam prison.

Another example in which results were translated in diverse actions is case 3: a study into resistance to anti-microbial drugs in Ghana. The study, which was initiated by a microbiology professor from a medical school, showed an alarming resistance to commonly used antimicrobials, such as tetracycline (82%), ampicillin (76%) and chloramphenicol (75%), and widespread multi-drug resistance [34]. The researchers provided several recommendations, such as training laboratory technicians, re-evaluating criteria for the use of antibiotics, enforcing laws on the sale of antibiotics and educating the public about their use. While participants described several plans that were inspired by the findings, most were shelved due to a lack of resources. The head of the Reference Laboratory described a plan to train laboratory technicians, but soon after this he retired. His successor was aware of the results, but did not mention any training plans and pointed to the lack of funding for such initiatives. The head of the Quality Assurance Unit described ideas to encourage laboratory testing before prescribing antibiotics, but had not taken any action. After additional interviews, two translation processes were identified. In response to feedback from the study, hospitals had taken the initiative to start a training for laboratory technicians. A policymaker pointed out that the results also played a role in discussions and decision-making about the list of essential medicines at the Ghana Health Service. This claim was confirmed by second interviewee who attended the same meeting.

Participants reported that the results of seven studies (cases 4, 5, 7, 23, 25, 27, 29) contributed, in diverse ways, to the design and implementation of the National Health Insurance Scheme. The development of the health insurance law was a lengthy, complex and sometimes highly contested process in which numerous actors were involved who negotiated about different proposals and plans, which slowly converged into the law that was eventually passed by Parliament in 2003 [35]. During this process, countless ideas, recommendations and plans were put forward in which all kinds of knowledge claims, experiences and interests played a role. Participants described how results of two studies were used in this process to support new proposals and challenge existing plans that were being developed. Case 5 showed that citizens wanted to be able to opt out of the insurance and districts needed additional funding to start-up the health insurance, which were both taken into account in the eventual policy. Participants described how the results of case 5, together with those of case 4, supported the choice for a district wide organization of the health insurance and provided insights in how these could be implemented. Case 4 also provided a method for identifying the poorest of the poor, which was adapted and then used in practice.

Other studies were used in the implementation of the National Health Insurance Scheme. The results of case 27 were used to successfully advocate for a flat fee system for reimbursing hospitals. Unit-cost data that were developed during this study were used by the Ghana Health Service to fund hospitals. The results of cases 7, 23 and 25 were used to improve the implementation of the insurance at district level. Case 7 showed local policymakers which groups were less likely to enroll in the insurance, after which a targeted enrolment campaign was organized. The results of both cases 7 and 25 helped to identify existing structures and networks through which the insurance could better reach target groups and collect premiums.

### Process features that were associated with the use of research

Below, we describe which features of research and translation processes were associated with the use of the produced knowledge. We start with the ones directly linked to the research program.

#### Fit with the national research agenda

The national health research priority strategy, which was a key component of the research program, helped to attune the research projects to the health sector priorities. The priority setting process, which interviewees described as useful, resulted in a research agenda with four priority themes (Box 1). These four themes matched with the health policy priorities that were described in the 2001–2006 Ghanaian Health Sector Programme of Work. The research agenda clearly influenced the formulation of research proposals. Some researchers said that they took the priorities as starting point for formulating a proposal. Others adapted their existing ideas and proposals to make them fit with the research agenda. Of the 30 assessed studies, 28 were clearly in line with the research agenda. This is unsurprising, since alignment with national needs was an important consideration in the selection of studies for funding.

**Box 1:** The priority themes of the national research agenda in Ghana

1) Communication and community participation Specific needs: health education approaches in Ghana, beliefs relating to health and prevention, evaluation of existing communication approaches and related interventions in the field of the Priority Health Service Interventions, piloting community involvement in policy formulation, planning, implementation and evaluation at district level and institutionalizing community involvement 2) Quality of health-care Specific needs: staff attitude, referral system, assurance of technical skills of providers, drugs and logistics management and monitoring and confronting anti-microbial resistance 3) Financing of health-care Specific needs: managing internally generated funds, improving management, formal and informal charges, pricing of drugs and services, introducing standardized pricing, comparative prices in private and public sectors, exemptions, especially for the poorest and most vulnerable, and culturally and gender-sensitive mechanisms to target the truly indigent and most vulnerable clients 4) Decentralization of health-care Specific needs: multi-sector coordination, integrating funding, balancing national and local priorities	

### Initiation by potential key users

Eighteen studies were initiated by people who were primary decision-makers or held influential positions in the health system (Table 2). These people defined research questions that arose from the programs they ran or advised and were thus themselves a potential key user. As one PI described: “*The proposal grew out of observations as a district director that there is a problem with the functioning of this level in the health system. From years of problems. All kinds of problems. Then you realize that, because when you talk with your other colleague district directors, and they all say yes, we also have this problem. So then it is like, instead of investigating this felt need in my little district, why don’t I look at it beyond. So it was a national scale study.*”

**Table 2:** Studies initiated by potential users

	User-initiated (n = 18)	Not user-initiated (n = 12)
Used (n = 20)	17	3
Not used (n = 10)	1	9

Examples of these ‘user-investigators’ include the head of the regional tuberculosis program who initiated a study into therapy adherence, the district director who aimed to better implement the vaccination program, and a member of a health financing committee who studied ways to fund hospitals. “*It very much influenced how the proposal was structured, because I realized there was a gap that needed some kind of investigation, some kind of evidence, to be able to present, if I should say, a paper for policy decision to be taken.*”

User-investigators were a striking feature of studies that were utilized: 17 of the 18 studies with a user-investigator were translated into action.

### Involving potential key users during the formulation of a research proposal

In addition to the potential key users who were part of the study team (as user-investigators), studies could also involve external potential key users during the formulation of the research proposal (Table 3). Participants described different reasons for consulting these external key users. Some were consulted to inform them about the proposal, ask for input, or increase the likelihood of use. Others mentioned that these potential key users had to be consulted in order to access to study populations, clinics or hospital administrations.

**Table 3:** Involvement of external users in developing the proposal

	User-initiated (n = 18)		Not user-initiated (n = 12)	
	No external users	Involved external users	No external users	Involved external users
Used (n = 20)	10	7	1	2
Not used (n = 10)	0	1	7	2

These consultations often led to adaptations of research proposals. The proposal of the study into maternal mortality was adapted after discussing it with the regional director: “*It was his idea that I should refocus on the service delivery factors, because that is what we have immediate control over. I had to remodel the framework a bit. I was going for a broader investigation”*. In the prison health study, the director of the Prison Service asked the researchers to include not only inmates, but also prison officers in the disease surveillance study.

User-initiators also discussed their proposals with other potential key users. One of them said: “*What changed the proposal? For example, comments like, well, because it is possible, we are going to look at this to inform policy in the whole health sector. The Ghana Health Service, which has over two hundred hospitals and over a thousand clinics. Can you expend the sample size? I think to about two of each type, across the country, about eight or so. Try to cover all types, locations? So that influenced the design and also the sample size.*”

External potential key users were involved in the formulation of eight of the 18 user-initiated studies and in four of the other 12 studies, of which two were used. In a further four studies, none of them were user-initiated; external key users were informed about research proposals, but were not involved in shaping them.

### Introducing new practices as part of research

Activities that were part of the implementation of the research itself could also contribute directly to action, and make it easier to use the results. In several cases, investigators and others provided examples of direct contributions that resulted from research activities, such as training health workers to follow a protocol, reorganizing administrative or logistical procedures, or teaching community members about HIV or the right to exemptions during interviews. While these direct contributions were of limited scope, interviewees said that they often remained after a study ended, and facilitated the use of the results. Case 12 provides a clear example. For the purpose of the study, new quality indicators were developed and teams were trained to use them to monitor quality of services in local clinics. After the study showed that this quality improvement strategy was beneficial, the use of these indicators was institutionalized in the involved clinics.

### Involving potential key-users in developing recommendations

In almost all used studies, potential key users were engaged in interpreting the meaning of the results and developing recommendations for action (Table 4). In 15 of the 18 user-initiated studies and five of the 12 other studies, external potential key users were involved in developing recommendations. A user-investigator said: “*First we sat down together in the region and pooled the study findings. We came out with an operational document. What is the job description of a sub district head? What support must be given to them? How should they relate? We then send it out, everybody has commented on it. We then said ok, let’s start working with this.*”

**Table 4:** Involvement of external users in interpreting results and developing recommendations

	User-initiated (n = 18)		Not user-initiated (n = 12)	
	No external key users	Involved ext. key users	No external key users	Involved ext. key users
Used (n = 20)	3	14	0	3
Not used (n = 10)	0	1	7	2

### Targeted distribution of printed results

The results of almost all studies were distributed in printed form beyond the scientific domain. In three cases, this dissemination was organized by the secretariat of the research program. In the other cases, the researchers had themselves taken the initiative to disseminate their results. Investigators who tried to mobilize others to use their results more often said that they adapted texts and prints to their target audience and sent it specifically to them: “*I send it to the Director of Human Resources and the Director General. What I did, I send not a research report, sometimes when people are busy, they don’t want a research report, but rather a memo.*”

Another investigator, who seemed very keen on the use of their results, described: “*When we did the final report. The Health Summit, you know the annual health summit. It was going on. We couldn’t get a slot to present the report, but were allowed to give people copies. So we carried copies of the report there and gave everybody a copy. We budgeted to print the report so that it looked attractive.*”

While the distribution of printed results may have supported translation processes, it was never described as playing an influential role in the use of research.

### The translation of results into action: examining the process

As the preceding paragraphs show, many researchers made concerted efforts to involve potential users in interpreting study results, and to make sure users were aware of those results. On further examination, we found that the translation of results into action involved a complex interplay between different actors with different ideas about the meaning of the results, actual change efforts in which results were used and evolving dynamics and structures in the context. Here, we describe some of these processes in more detail.

### Envisioning what should be done and who should do what

Researchers were themselves the first to shape the meaning of their study results. In four of the cases we studied, the investigators said that their results had no immediate implications for action. These investigators argued that their results confirmed existing knowledge or that further research was required. One of them explained: “*My findings and recommendations are not new things to the people in policy. They are things they already know. If there is anything at all, the presentation would only be to reinforce, to tell them that what they are doing is in the right direction.*” Not surprisingly, the results of these studies were not used to contribute to changes in policy or practice.

In the other 26 cases, the investigators said that their results should be translated into action, and they had ideas about how that should happen, and who should be involved. To achieve the changes they envisioned, actors put forward more or less explicit stories about a desired future, in which they assigned roles and responsibilities to a variety of actors and described what they should be doing. Depending on the forces at play and the situation in which these ‘actor-scenarios’ were put forward, research knowledge was assigned a role in them.

A technical advisor who aimed to use the results of the antimicrobial resistance study provides an example of a scenario of the future in which roles and responsibilities were assigned to several actors: “*The results show the Ministry of Health that what is happening in Accra is going on all over the country. From now on, the Regions must apply the law. The Ministry must take the results and use them to educate the pharmacists. They need to better explain how to take the medication. They must also educate the general population and thirdly, the herbalist who mix antimicrobial agents with their herbs. They have to stop that.*”

The stories about what results meant for action were not automatically accepted. Some people became inspired and put forward similar or somewhat modified actor-scenarios. Others started to resist the envisioned futures and roles they were assigned, and put forward alternative views in which the results had different implications for what should be done and who should do what. This could lead to further actions and interactions, after which a relatively stable set of ideas emerged about what results meant for action.

We analyzed who, according to the investigators, should play a role in the scenario’s which they described as leading to change (Table 5). In 14 cases, the investigators said that they should themselves play a key role in achieving change. In the other 12 cases, the investigators envisaged others playing the main role.Investigators gave different reasons why others had to play a key role in applying their results. Eight of them said they were constrained because they did not work in the health sector. For some, this was reason enough not to foresee a role for themselves in acting on the study results. “*I am an economist and I work here at the university. We presented to them* [involved hospitals] *and gave them the report* […] *How far they took it? It is up to them to use the result or not.*”

**Table 5:** According to the investigators: who should play a key role in using the results to achieve change?

	User-initiated (n = 18)	Not user-initiated (n = 8)
Others should play key role in achieving change (n = 12)	4 (3 used)	8 (3 used)
Investigators should play key role in achieving change (n = 14)	14 (14 used)	0

Not all investigators shared this idea. Three of the eight, also university-based researchers, saw a role for themselves even though they did not imagine that they would be the prime movers in achieving change. They described a strong motivation to encourage others to use their results for change. One of them explained: “*We did the study so it is logical for us to want to move the findings forward. I am not sure if anyone else would try to move the findings forward*.”

Others said that they would not be able to take forward their results because they lacked the required seniority, influence or responsibility. One investigator said that, in Ghanaian culture, he would be considered too young to advise policymakers. Another investigator who was a district director and keen on the use of his findings, nonetheless felt that the results should be used in national policy processes, to which he had limited access and which were going in a different direction than the recommendation of his study.

### Who mobilized results to achieve change?

Once the implications of results for action became accepted, people drew upon this accepted knowledge, and were influenced by it, to make real changes in policies and programs (Table 6). In 14 of the 20 used studies, one of the user-investigators played a key role in using this knowledge for achieving change. In the other six studies that were used, others, with whom the results had been personally discussed, played a key role in making change happen. In only one case we identified a translation process that happened without any interaction with the investigators, but this occurred more than 2 years after a study ended.

**Table 6:** Who played a key role in using results to achieve change where study results were used?

	User-initiated (n = 17)	Not user-initiated (n = 3)
User investigator	14	–
External user involved since formulation	2	1
External user involved only in interpretation	1	2

In all our cases, translations required efforts or support from people with a specific formal position, such as a regional health director, a program manager or a working group at the Ministry of Health. These formal positions were described as essential for acquiring support, mobilizing resources and making new knowledge part of concrete policies and practices.

Several investigators described how their position in policy processes became more influential because they were conducting a study. One of them said: “*When you do the study, then they know, it gives you a kind of authority in that area, they listen because you are involved, you have the data*.”

In addition, some investigators said that they could scale up the use of their results when they themselves shifted positions, usually through promotions. An example is the district director in the immunization case, who was promoted to regional director during his study, and then transferred to lead a new region: “*When I came to this region,* […] *I found out that most of the things I saw over there, I am seeing here. I am carrying my luggage with me. Wherever I am going, the data goes with me*.”

Interviewees pointed out that formal positions also had their limitations. Their influence was limited to specific subject areas, locations and directions. They also emphasized that trust, reputation, advocacy skills and sheer persistence could be just as important for gaining access to policy arenas, gathering support and mobilizing resources so that results could be turned into action.

### The role of structures and dynamics in the context

Translations were not only shaped by actors and the coalitions they build, but also by the evolving world in which processes were embedded. Ideas, budgets, local practices, equipment and infrastructures that played a role in the envisioned change and in concrete actions could not be mobilized at will, but were entangled in a larger world full of existing structures and ongoing dynamics. Ideas were linked to value systems, budgets were part of financing schemes, practices were embedded in a social order, equipment depended on trained health workers and physical infrastructures were shaped by the local landscape. As a result of these entanglements, the structures and dynamics in the larger world enabled some translations and constrained others.

While we focused our analyses on the actions of individuals, in some cases, these structures and dynamics seemed just as important for how translations worked out. An example of a larger dynamic that influenced several translation processes was the design and implementation of the National Health Insurance Scheme. Cases 5 and 6 illustrate how this interacted with research and translation efforts. Case 5 examined the functioning of district level health insurance schemes, while the study in case 6 focused on community based health insurance. The study in case 5 was formulated and executed in relative isolation, with no involvement of potential key-users. Study 6 was led by a district health director, who interacted with potential key-users and was keen on making a contribution. When the national task force, which designed the blueprint for the National Health Insurance Scheme, was considering the role of the districts, some members became very interested in the recommendations from case 5. “*We had just finished the project when the government wanted to adapt the health insurance program. Most of our recommendations were incorporated in what was eventually adopted as national policy*.” Despite the intentions and position of the district director, the results from case 6 were neglected by the national task force: “*It has not contributed to national policy because it didn’t fit the current agenda. It should have, and I think it is a prophecy document*.”

## Discussion

The aim of our study was to map the contribution of health research to action and examine which features of research and translation processes were associated with the use of the results. All 30 cases in our sample were part of a program of health research which aimed particularly to foster locally led, demand-driven studies in Ghana.

Overall, we found that, in 20 of the 30 assessed research projects, a contribution to action for health could be identified between 6 and 12 months after studies were finalized. It is difficult to compare this use rate with other research programs, since data are sparse. The few studies that have been published tend to focus on a small number of cases, use self-reporting without triangulation, and/or interview a limited number of informants [13, 36, 37]. Perhaps the most similar study to ours was recently conducted in Australia, and used a questionnaire, one interview per case and a panel to assess the “*real world policy and practice impacts*” of 50 intervention studies within 5 years after finalization. In this study, 38% of the cases seemed to have ‘impact’, though this could not always be corroborated [15]. In our study of 30 cases, for which we interviewed several informants per case, the results of 67% of the studies were used to contribute to action for health within a year.

Our analysis suggests that this relatively high proportion is related to the strategy of the research program, which was designed specifically to enable studies that would be likely to contribute to action. Two aspects in particular seem to have made a difference. The first was the process of priority setting and study selection, which led to the funding of studies that were, from the onset, closely aligned with local health sector priorities, and that therefore posed questions that met the immediate information needs of those who shaped policy in health.

The second, and perhaps most important aspect of the program strategy in terms of the eventual use of research results, was that research had to be initiated and led by Ghanaians and that health sector professionals as well as academics were eligible to initiate studies.

Looking more closely at which features of research were most strongly associated with eventual use of study results, we found that one stood out above all. That was the presence of a single person who initiated the study, remained involved in the process, and was in a position to use the results in their own work. Critically, these user-investigators were likely to formulate ‘need-to-know’ research questions that filled urgently-felt information gaps and took initiatives to use their result. The results of 17 out of 18 of the studies involving user-investigators were translated into action.

The use of the results by the user-investigator was not the only reason why 17 of these 18 studies were used. Studies initiated by potential end-users were also more likely to involve other potential users in the formulation of research as well as in interpreting results and developing recommendations. In three cases, the user-investigators themselves did not have a major role in using study results. It was their ongoing interaction with other ‘external’ potential users that seemed to enable the use of the results.

If none of a study’s investigators was themselves a potential key user, interaction with external potential users seemed critical to the use of results. In one case, involving research in prisons, potential key-users contributed to both the study design and the interpretation of results. In three other cases, potential key-users were not involved until the field work was completed. It was their engagement in the interpretation of study results that appeared to contribute to the translation of research into action.

Our findings are in line with an analysis of research impact in the United Kingdom by Greenhalgh and Fahy [38] in which the use of research was characterized by an ethical commitment by researchers, strong institutional support and a proactive interdisciplinary approach to impact activities. Our findings contradict attempts to explain the use of research in terms of the characteristics of the results, such as their salience, applicability or validity [39–41]. While results could certainly play a role, we found that the use of research was strongly influenced by those who put forward what the results meant for action. The involvement of potential key users in this process seemed to contribute to developing recommendations and concrete plans that were broadly feasible, taking into account the validity of results, the specifics of the local situations and the aims of those who shaped policies for health.

Our analysis of how translation processes evolved suggests that there were two overall dynamics in the translation of knowledge into action: a first, in which investigators and others put forward stories about what results meant for action, which, after interaction and stabilization, could become part of the repertoire of locally accepted knowledge; and a second dynamic, in which actors drew upon this accepted knowledge, and were influenced by it, to actually effect change. These processes were not linear or isolated, but recursive, and embedded in, and interacting with, ongoing action and dynamics and structures in the context [42].

This perspective on knowledge translation may be useful to those who study how context influences the use of research, evidence briefs or other knowledge products [43, 44]. The actor-scenario perspective suggests that context cannot easily be studied as a set of fixed factors that somehow have effect on the use of research, as different users may put forward very different actor-scenarios, in which the same results play a very different role and very different elements of ‘context’ are mobilized.

While several studies in other countries find that interaction with users enhances the likelihood that research is used, this is the first study to our knowledge in which the relation between what happens throughout research processes, and the use of the results, is systematically analyzed in a substantial number of demand-driven, locally led studies in a lower-income country.

Our findings support those of Walley et al. [45], who, based upon experiences in China and Pakistan, argued for an approach of “*getting practice into research: to get research into practice*”, especially for operational research in developing countries. An advantage of such an approach is that a problematic gap between researchers who ‘discover’ and policymakers and practitioners who ‘apply’ may not emerge [46]. Such an approach is perhaps not appropriate for research into new and untried treatments were the efficacy has not been established, but our study shows that it has great application for applied research.

A possible limitation of the focus on potential key users is that the use of results could be constrained by their authority or influence. In our study, we found this not generally to be the case. In part because they were promoted and transferred, and in part because the use of results created a concrete example to others, which helped to spread changes more widely. A potential risk of involving influential users in research processes is that their aims and interests may bias research. While this requires attention, it is important to recognize that researchers themselves have their own aims, interests and perspectives, which may also need to be reflected upon [23].

Researchers tend to focus on the written texts that they produce and disseminate and which they hope are picked up by others and then translated into action. We observed that results were mostly spread by people who were moving about, personal interaction and through the spread of successful innovations in which results were used. In none of our cases was the dissemination of written texts described as important for the use of results. This can be explained perhaps by the important role of user-investigators and personal interaction, which may have replaced the role of written texts, our selection of interviewees, and our 1-year follow-up, which seems short compared to other studies. Another explanation is that the use of written texts by unknown individuals, at unknown times and places is rather difficult to map, which may lead to an overestimation of the role of interaction [7].

This study shows both the potential and importance of locally led, demand-driven health research in lower-income countries. The approach of the research program was inspired by the critique in the early nineties that health research contributed little to health and development in poorer countries because it was dominated by foreign scholars instead of locally embedded researchers, and met international rather than local needs. The research program tried to turn this around by fostering research that was driven by local demands and led by local researchers. Our analysis shows the success of this approach in terms of contributing with research to action for health. This finding corresponds with analyses of research programs by others in several other countries [47, 48]. Our analysis also shows the importance of local research for improving local action for health. While the studies did not produce major scientific breakthroughs, they often played a key role in improving local action for health, which is remarkable given their small budgets.

### Considerations for research policy

The results allow us to formulate some suggestions for those who attempt to support research that more effectively contributes to health in low-income countries and elsewhere.

A first suggestion is to continue promoting national research priority setting, which is becoming increasingly common around the world [49, 50]. While priority setting is only a first step, a demand-driven priority agenda can assist researchers in formulating proposals towards local needs and can help funders to select studies that are more likely to be used. A careful and inclusive priority setting process not only helps to orient research to needs, but also provides a platform for interaction, building trust and networking, which are important for the eventual use of research [50–52].

A second suggestion is to stimulate research that is initiated and conducted by those who can play a role in the use of the results. A challenge is that the number of professionals with an influential role in health policy, sufficient research skills and the necessary time for research is likely to be small [53, 54]. It may be worth exploring how these user-investigators can best be incentivized and supported in their work, for example, by junior researchers [55].

The third suggestion is to engage potential key users in research processes from the start, especially in designing research proposals, interpreting results and formulating recommendations. To select potential key users, researchers can try to envision how results may be used and who will play a role in that process (an actor-scenario), and then try to involve those who seem most interested and influential.

While this study shows the advantages of demand-driven research, several cases show that more independent and critical research is also essential for improving global health [56–59]. A risk of a unilateral focus on demand-driven research is that it may take prevailing ideas, power relations and dominant elites as a starting point, and may lead to ignoring questions about what dominant views are based upon, the effects of power relations, and the needs of more marginalized groups [25].

### Limitations

The detailed interviews showed that each case was unique, context-specific and far more nuanced that we have been able to describe in this paper. In order to assess what played a role in whether research was used or not, we have been obliged to reduce a precarious, ongoing and complex process into a snapshot of a limited period and number of actors and actions. The ‘use’ and ‘non-use’ of results, for example, actually covers a wide and dynamic spectrum which is not fully reflected in a binary categorization. Similarly, the roles of individuals in the inherently collaborative process of research did not always fit as neatly into binary categories of ‘user-initiated’ or ‘not user-initiated.’ Our iterative and inclusive research design aimed to minimize the subjectivity of these simplifications. The large number of interviews, openness of participants and the relatively small number of key actors involved in both the research and policy community helped us to examine how processes evolved and to triangulate claims. While some investigators had the initial tendency to under or overestimate the use of their research, the shared exploration of how processes evolved often helped to describe the role that research knowledge had played.

## Conclusions

In examining the contribution of health research to action we identified a number of features which have implications for organizations that support research, especially but not exclusively, in low- and middle-income countries. Our study underlines the importance of supporting research that meets locally-expressed needs and that is led by people embedded in the contexts in which results can be used. Supporting the involvement of health sector professionals in the design, conduct and interpretation of research appears to be an especially worthwhile investment.
